# An hnRNP-like RNA-binding protein affects alternative splicing by *in vivo* interaction with transcripts in *Arabidopsis thaliana*

**DOI:** 10.1093/nar/gks873

**Published:** 2012-10-05

**Authors:** Corinna Streitner, Tino Köster, Craig G. Simpson, Paul Shaw, Selahattin Danisman, John W. S. Brown, Dorothee Staiger

**Affiliations:** ^1^Molecular Cell Physiology, Bielefeld University, ^2^Cell and Molecular Sciences, ^3^Information and Computational Sciences, The James Hutton Institute, University of Dundee, ^4^Division of Plant Sciences, University of Dundee at the James Hutton Institute, Invergowrie DD2 5DA, Scotland, UK and ^5^Institute for Genome Research and Systems Biology, CeBiTec, D-33615 Bielefeld, Germany

## Abstract

Alternative splicing (AS) of pre-mRNAs is an important regulatory mechanism shaping the transcriptome. In plants, only few RNA-binding proteins are known to affect AS. Here, we show that the glycine-rich RNA-binding protein *At*GRP7 influences AS in *Arabidopsis thaliana*. Using a high-resolution RT–PCR-based AS panel, we found significant changes in the ratios of AS isoforms for 59 of 288 analyzed AS events upon ectopic *At*GRP7 expression. In particular, *At*GRP7 affected the choice of alternative 5′ splice sites preferentially. About half of the events are also influenced by the paralog *At*GRP8, indicating that *At*GRP7 and *At*GRP8 share a network of downstream targets. For 10 events, the AS patterns were altered in opposite directions in plants with elevated *At*GRP7 level or lacking *At*GRP7. Importantly, RNA immunoprecipitation from plant extracts showed that several transcripts are bound by *At*GRP7 *in vivo* and indeed represent direct targets. Furthermore, the effect of *At*GRP7 on these AS events was abrogated by mutation of a single arginine that is required for its RNA-binding activity. This indicates that *At*GRP7 impacts AS of these transcripts via direct interaction. As several of the AS events are also controlled by other splicing regulators, our data begin to provide insights into an AS network in *Arabidopsis*.

## INTRODUCTION

Pre-mRNAs arising from the same genomic locus can be processed into multiple transcript isoforms by variable use of splice sites, combining different regions of the transcripts (1). This process, alternative splicing (AS), increases proteome complexity through the generation of protein isoforms with variable domain composition from pre-mRNAs from the same gene. AS also regulates expression at the level of transcript stability, where AS can generate transcripts with premature termination codons (PTCs) that are degraded via the nonsense-mediated decay (NMD) pathway (2).

In metazoa, *cis*-regulatory elements have been identified (splicing enhancers and splicing suppressors) that are located in exons or introns and influence AS through interaction with *trans*-acting proteins to determine splice site choice and where the spliceosome will assemble (3). The main families of splicing factors are the serine/arginine-rich (SR) proteins and heterogenous nuclear ribonucleoproteins (hnRNPs). SR proteins are characterized by one or two RNA recognition motifs (RRMs) and the SR domain that engages in protein–protein interactions (4,5). hnRNPs contain diverse types of RNA-binding domains including RRMs and KH domains (6,7). These AS regulators either activate or repress selected splice sites dependent on the position of their binding site and interactions with other factors (8). In addition to these general regulators, specific proteins affect AS of a defined subset of transcripts (9).

In the higher plant *Arabidopsis thaliana*, 61% of intron-containing genes undergo AS under regular growth conditions (10). AS is influenced by environmental factors including temperature stress and pathogen attack (11–13) and therefore this number is likely to be a lower limit. Intron retention is the most common AS event accounting for 40–50% of the AS events in *Arabidopsis*; in humans only 5% of the AS events correspond to retained introns (14–17). However, recent analysis of AS in *Arabidopsis* showed that on a transcript level rather than the level of individual AS events, intron retention had much less impact reflecting the likelihood that many annotated intron retention events are derived from partially spliced transcripts (10). Skipping of entire exons is relatively rare in *Arabidopsis* (8%), whereas it accounts for 58% of AS events in humans (18). This fundamental difference in the prevalent types of AS events is thought to reflect differences in gene structure (plant introns are generally much shorter than introns in animals) and in the way plants recognize introns (plant introns are often UA-rich). Our knowledge of sequence motifs that influence the choice of splice sites and the cognate protein factors is still limited and a comparative analysis of splicing regulators in plants is of major interest.

The best studied plant splicing factors are the SR protein and polypyrimidine tract-binding protein (PTB) families (5,19,20). Ectopic expression of some of the 18 SR proteins in *Arabidopsis* produces a range of morphological and physiological phenotypes and impacts AS of their own pre-RNAs and of a suite of other transcripts (21,22). To date, several hnRNP-like proteins have been identified in plants but an involvement in splice site control is not well documented (23). UBP1 (oligouridylate-binding protein 1) from *Nicotiana plumbaginifolia* is a nuclear RBP that binds to U-rich sequences in introns and untranslated regions (UTRs) (24). It enhances the splicing efficiency of otherwise inefficiently processed introns and increases steady-state abundance of reporter mRNAs that have either no or suboptimal introns. A related hnRNP-like protein RBP45 from *N. plumbaginifolia* also enhances intron recognition of a mini-exon reporter (25). The hnRNP-like PTB1 and PTB2 proteins show negative auto- and cross-regulation by AS of their own pre-mRNAs, where inclusion of a PTC-containing exon creates an NMD substrate (19). Downstream targets have not been identified as yet.

Plants also contain a family of small hnRNP-like glycine-rich RBPs. The *At*GRP7 (*A. thaliana* glycine-rich RNA-binding protein 7) and *At*GRP8 proteins have been linked to AS. Both contain a single RRM-type RNA-binding domain with the highly conserved RNP-2 and RNP-1 motifs and a glycine-rich stretch, and are under control of the circadian clock (26–28). Through reverse genetics, *At*GRP7 has been shown to be part of an auto-regulatory feedback loop (29). Ectopic over-expression of *At*GRP7 in transgenic plants leads to the use of an alternative 5′ splice site in the intron of the endogenous *AtGRP7* pre-mRNA. The resulting AS isoform retains part of the intron with a PTC and is short-lived. Its degradation depends on UPF1 (UP FRAMESHIFT PROTEIN 1) and UPF3 that are part of the NMD pathway (30,31).

In this study, we show that *At*GRP7 has a more global effect on AS. For this, we employed an RT-PCR system designed to analyze known AS events with high sensitivity and high resolution (10,32–35). We identified splicing events that are controlled by both *At*GRP7 and its paralog *At*GRP8. For several transcripts, the ratio of AS isoforms changes in opposite directions in plants constitutively over-expressing *At*GRP7 or in plants lacking *At*GRP7, respectively, suggesting that these transcripts may be direct *At*GRP7 targets. Indeed, we showed by RNA immunoprecipitation (RIP) from whole cell extracts that seven of the identified transcripts are bound by *At*GRP7 *in vivo*. Furthermore, the effect of *At*GRP7 on AS of some of these targets is abrogated by mutation of a single arginine within the RRM required for RNA binding and *in vivo* function (36,37) indicating that *At*GRP7 affects AS of these transcripts via direct interaction. Several of the AS events controlled by *At*GRP7 are also controlled by SR proteins or the cap binding complex, either in the same direction or antagonistically. Thus, our data begin to provide insights into an AS network in *Arabidopsis*.

## MATERIALS AND METHODS

### Plant material and growth

*Arabidopsis* seeds were surface-sterilized and sown on agar-solidified half-strength MS (Murashige-Skoog) medium (Duchefa) supplemented with 0.5% sucrose and 0.5 g MES/I (38). Plants were grown in 16-h light/8-h dark cycles at 20°C in Percival incubators (CLF laboratories). All above-ground parts of the plants were harvested after 2 weeks at zt10 (Zeitgeber time 10, 10 h after lights on) when *AtGRP7* level peaks. The genotypes used were Col-2 wild-type (wt), *At*GRP7-ox plants constitutively over-expressing *At*GRP7 under the control of the Cauliflower Mosaic Virus promoter (CaMV) in the Col-2 background (39), C24 wt, *At*GRP7-ox in the C24 background (40), *At*GRP8-ox constitutively over-expressing *At*GRP8 (31) under the control of the CaMV promoter in the Col-2 background, and *At*GRP7-RQ-ox plants constitutively over-expressing a mutated *At*GRP7 protein with arginine 49 exchanged for glutamine (R^49^Q) under the control of the CaMV promoter both in the Col-2 and C24 background (36)*.* A line without *At*GRP7 expression and with reduced *At*GRP8 levels was generated by crossing the *atgrp7-1 *T-DNA insertion mutant (41) to the RNA interference line *AtGRP8*i-l71 with an RNAi construct directed against *At*GRP8 (39). Homozygous F2 plants were identified and designated *atgrp7-1 8i*. Levels of *At*GRP7 and *At*GRP8 protein in each of the analyzed lines are shown in Supplementary Figure S1.

### RNA isolation and high resolution RT-PCR AS panel

Total RNA was extracted from above-ground tissue using TriReagent (GE Healthcare, Freiburg, Germany) and treated with the DNase kit (Qiagen, Hilden, Germany). RNA (5 µg) was reverse transcribed using M-MuLV RT and oligo(dT)_18_ primer. After inactivation of the enzyme, PCR was performed in a 96-well format (24 cycles). The forward primers were labeled with the fluorescent dye 6-carboxy fluorescein. Each RT-PCR reaction (1 µl) was diluted into 10 µl Hi-Di formamide (Applied Biosystems) and 0.05 µl GeneScan 500 LIZ internal size standard. The fragments were separated on an ABI3730 DNA Analyzer (Applied Biosystems) and analyzed using the GENEMAPPER Software (Applied Biosystems). The RT-PCR products with the sizes expected for the respective AS isoforms were identified. The percentage of each AS isoform relative to the sum of all relevant AS isoforms from each RT-PCR reaction was calculated using the fluorescent peak areas. The mean ratio of AS isoforms for each event was determined based on three biological replicates. Significant changes in the ratios of AS isoforms were identified from direct comparisons of values from wt and over-expression or mutant lines according to a *t*-test analysis (significance with *P* ≤ 0.05). We initially focused on transcripts that showed a significant increase or decrease with a minimum 5% difference between the means of wt plants and over-expression or mutant plants, respectively. However, some AS events showed significance even though the difference in ratios was 3–5%—the lower level was selected because we previously determined that different technical reps in the AS RT-PCR system showed a SEM of up to 3% (33).

The AS panel contains primers for *ACTIN11* and *RPL12c* that serve as reference transcripts to allow determination of relative expression levels of all splice forms. To calculate the total transcript level, the fluorescent peak area of all relevant AS forms was normalized to the level of *ACTIN11* and *RPL12c*, respectively. Supplementary Table S1 contains the complete list of genes and primer pairs used on the RT-PCR panel.

### RNA immunoprecipitation

*At*GRP7 was fused in frame to Green fluorescent protein (GFP) and expressed under the control of the *AtGRP7* promoter (42) and authentic *cis*-regulatory sequences within the transcribed part of the gene, i.e. 5′-UTR, 3′-UTR and introns, and introduced into *atgrp7-1*. As a control, transgenic plants expressing GFP only under the control of the *At*GRP7 promoter including 5′- and 3′-UTR were generated. Plants grown in long day conditions on agar plates for 2 weeks were vacuum-infiltrated with 1% formaldehyde for 15 min, followed by quenching with 125 mM glycine. A whole-cell extract was prepared in RIP-lysis buffer [50 mM Tris–HCl pH 7.5, 150 mM NaCl, 4 mM MgCl_2_, 0.1% Igepal, 0.1% SDS, 5 mM DTT, 10 mM vanadylribonucleosid complex, 100 U RiboLock™/ml (Fermentas), 1 mM phenylmethylsulfonylfluorid and protease inhibitor tablets (Roche)]. The extract was pre-cleared with Sepharose beads and subjected to immunoprecipitation with GFP-Trap® beads (Chromotek, Martinsried, Germany), hereafter called IP+. A mock immunoprecipitation was performed using Red fluorescent protein (RFP)-Trap® beads (Chromotek), hereafter called IP−. After extensive washing, coprecipitated RNAs were eluted with TriReagent from IP+ and IP− samples, respectively, and quantified in duplicates via qPCR essentially as described (43). In parallel, transcript levels were determined in RNA isolated from the extract (input). PCR primers are listed in Supplementary Table S1.

### Immunoblots

Preparation of protein extracts and western-blot analysis with antibodies against *At*GRP7 and *At*GRP8 were done as described (44,45).

## RESULTS

### Identification of AS events influenced by constitutive over-expression of hnRNP-like *At*GRP7

The hnRNP-like RBP *At*GRP7 auto-regulates AS of its own pre-mRNA and of its closest paralog *AtGRP8* (30,31). The impact of *At*GRP7 on AS of additional transcripts was examined using the high resolution RT-PCR AS panel described previously (33). A total of 278 primer combinations were used to analyze 288 AS events in transcripts encoding stress-related proteins, transcription factors, RNA-binding proteins, flowering time regulators and other proteins selected from published work or databases (33) (Supplementary Table S1). The splicing patterns in 14-day-old transgenic plants constitutively over-expressing *At*GRP7 (*At*GRP7-ox) were compared to those in Col-2 wt plants. In *At*GRP7-ox plants, a change in the ratio of splice variants >5% relative to wt plants was found for 59 events (21%) (*P* < 0.05) (Supplementary Table S2). Of these, 27 events were alternative 3′ splice sites, 24 were alternative 5′ splice sites, 7 events were exon skipping and 2 were intron retentions (Table 1). Forty-one percentage of the AS events affected by *At*GRP7 over-expression were alternative 5′ splice sites including *AtGRP8* AS at a cryptic 5′ splice site within its intron (30,31). Considering that only 23% of all AS events on the panel represent alternative 5′ splice sites, this indicates that *At*GRP7 preferentially influences the choice of 5′ splice sites (*P* = 0.0004 determined by hypergeometric test). Only 3% of the events influenced by *At*GRP7 represented intron retention, in contrast to 17% present in the panel (*P* = 0.0006 determined by hypergeometric test).

**Table 1.** Types of AS events showing significant changes in *At*GRP7-ox plants in the Col-2 background

Types of AS events	288 Events analyzed		59 Events changed in *At*GRP7-ox (Col-2)	
	No.	%	No.	%
Alternative 3′ splice site	135	47	26	44
Alternative 5′ splice site	67	23	24	41
Intron retention	48	17	2	3
Exon skipping	34	12	7	12
Cryptic intron	4	1	0	0

The ratios of AS forms could change due to regulation of splice site usage by *At*GRP7 or change indirectly as a consequence of altered steady-state abundance of individual AS forms. Therefore, transcript levels of all investigated splice variants were calculated relative to *ACTIN11* and *RPL12c*. In the *At*GRP7-ox plants, 12 of the 278 transcripts analyzed (4.3%) showed a difference in steady-state abundance of at least 2-fold (Supplementary Table S3). Of the 59 transcripts with a change in AS in *At*GRP7-ox plants, only 4 also showed an altered steady-state abundance of at least 2-fold (6.8%). Thus, *At*GRP7 over-expression does not significantly alter steady-state abundance of the affected transcripts (*P* = 0.1501 determined by hypergeometric test). We conclude that the observed changes in the ratio of individual splice variants are due to changes in splice site selection as a consequence of *At*GRP7 over-expression.

**Figure 1.**
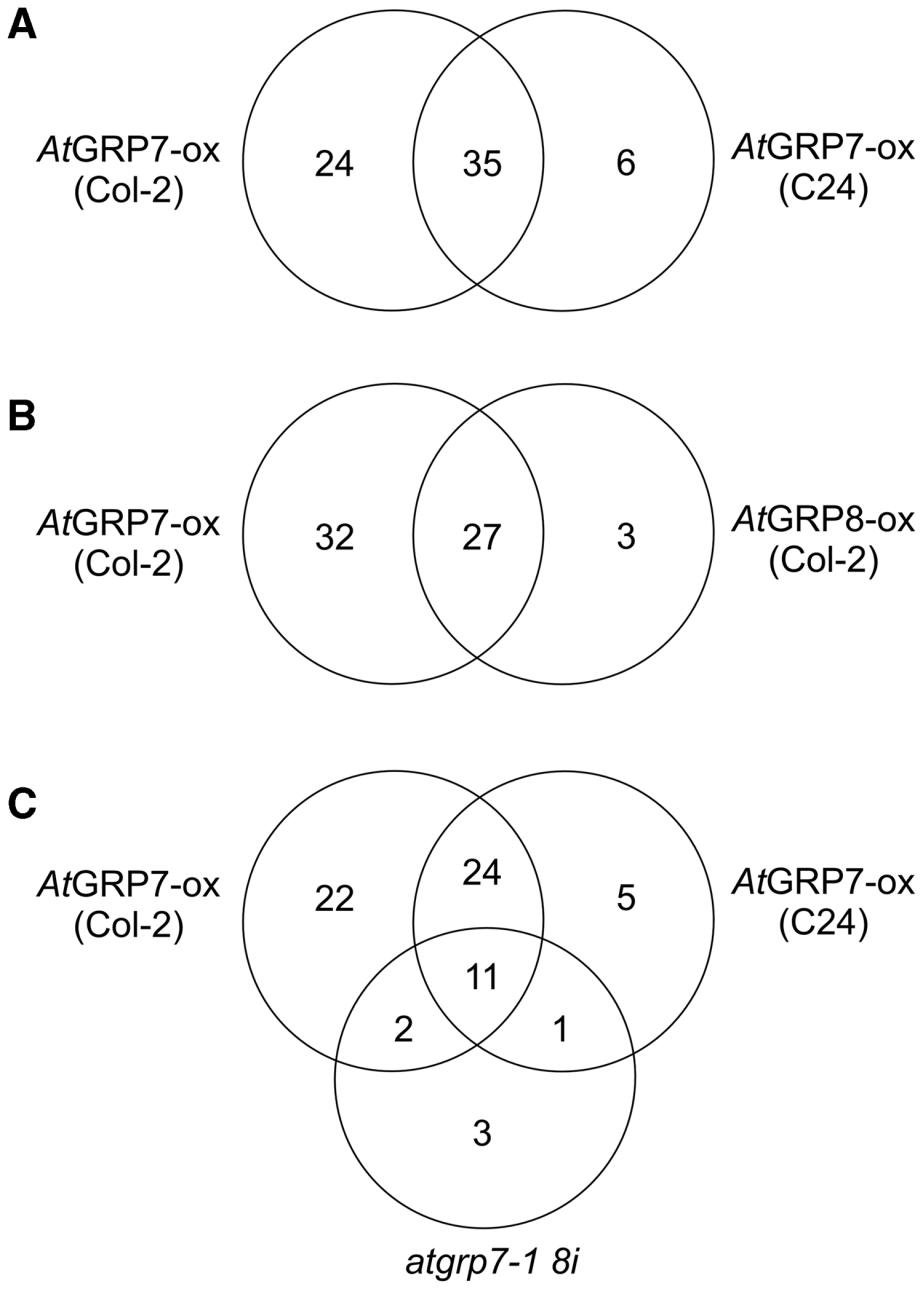
Changes in the ratio of AS isoforms in *At*GRP7-ox, *At*GRP8-ox and *atgrp7-1 8i*. (**A**) Venn diagram showing the number of splicing events with significant changes in *At*GRP7-ox (Col-2) and *At*GRP7-ox (C24) when compared to the Col-2 and C24 wild-types, respectively. (**B**) Venn diagram showing the number of splicing events with significant changes in *At*GRP7-ox (Col-2) and *At*GRP8-ox (Col-2). (**C**) Venn diagram showing the number of splicing events with significant changes in *At*GRP7-ox (Col-2 and C24) and *atgrp7-1 8i*. The numbers represent transcripts with significant changes in the ratio of AS isoforms (>5% and *P* < 0.05).

To increase confidence in the effects of *At*GRP7 on AS, we tested the influence of *At*GRP7 in a different genetic background. In an independent *At*GRP7-ox line in the C24 background (Supplementary Figure S1), 41 of 87 events analyzed [(including the events most strongly affected in AtGRP7-ox (Col-2) plants)] were changed significantly compared to C24 wt (>5%; *P* < 0.05) (Figure 1A). Of these, 35 were also changed significantly in *At*GRP7-ox (Col-2) (Table 2). This supports the conclusion that elevated *At*GRP7 levels are responsible for the changed ratios in AS isoforms in the *At*GRP7-ox lines.

**Table 2.** Genes/transcripts with significant changes in the ratio of alternatively spliced isoforms in *At*GRP7-ox plants

primer pair	AGI	Description	Product sizes (bp)	Col-2		*At*GRP7-ox (Col-2)		*P*-value	C24		*At*GRP7-ox (C24)		*P*-value
				Small prod. (%)	Large prod. (%)	Small prod. (%)	Large prod. (%)	7-ox (Col-2)	Small prod. (%)	Large prod. (%)	Small prod. (%)	Large prod. (%)	7-ox (C24)
Alternative 3′SS													
12	At1g72320	APUM23 (Arabidopsis pumilio 23); RNA binding	141/150/215	15	83	5	95	0.0002	23	74	8	92	0.0192
19	At2g32320	tRNA His guanylyltransferase	202/273	33	67	45	55	0.0047	31	69	39	61	0.0048
36	At4g12790	ATP-binding family protein	212/338	45	55	25	75	0.0029	46	54	25	75	0.0034
49	At5g41150	UVH1 (ultraviolet hypersensitive 1)	293/346	82	18	72	28	0.0003	92	8	84	16	0.0034
59	At5g66010	RNA binding	105/182	30	70	40	60	0.0261	38	62	59	41	0.0016
242	At1g60850	ATRPAC42 (*Arabidopsis thaliana* RNA polymerase I subunit 42); DNA binding/DNA-directed RNA polymerase	111/122	70	30	77	23	0.0311	73	27	79	21	0.0325
254	At5g50240	l-isospartyl methyltransferase	192/201	63	37	73	27	0.0282	64	36	73	27	0.0079
273	At3g07810	Heterogeneous nuclear ribonucleoprotein, putative/hnRNP, putative	126/173	8	92	3	97	0.0094	10	90	4	96	0.0372
288	At3g12570	FYD	159/188	43	57	52	48	0.0148	39	61	53	47	0.0207
295	At2g02390	ATGSTZ1 (glutathione s-transferase 18); glutathione transferase	181/202	88	12	69	31	0.0023	87	13	71	29	0.0016
378	At3g62190	DNAJ heat shock N-terminal domain-containing protein	144/334	89	11	99	1	0.0075	85	15	98	2	0.0032
Alternative 5′SS													
72	At2g04790	Similar to unnamed protein product [Vitis vinifera] (GB:CAO23994.1)	167/190	68	32	53	47	0.0077	41	59	29	71	0.0027
75	At2g36000	Mitochondrial transcription termination factor-related/mTERF-related	150/254	31	69	14	86	0.0009	40	60	17	83	0.0016
87	At4g35450	AKR2 (ankyrin repeat-containing protein 2); protein binding	305/350	78	22	59	41	0.0052	72	28	57	43	0.0009
90	At4g39260	ATGRP8/GR-RBP8 (CCR1)	158/316	94	6	23	77	0.0000	97	3	23	77	0.0004
112	At1g09530	PAP3/PIF3/POC1 (phytochrome interacting factor 3); DNA binding/protein binding/transcription factor	230/290	88	12	59	41	0.0000	90	10	66	34	0.0033
129	At2g40830	RHC1A (RING-H2 finger C1A); protein binding/zinc ion binding	220/329	91	9	59	41	0.0012	91	9	63	37	0.0037
136	At3g07740	ADA2A (Arabidopsis adaptor 2A homolog); DNA binding/transcription factor	139/240	100	0	46	54	0.0006	100	0	70	30	0.0063
141	At3g51880	HMGB1 (High mobility group B 1); transcription factor	204/225	92	8	79	21	0.0471	92	8	87	13	0.0003
145	At3g17609	HYH (Hy5-homolog); DNA binding/transcription factor	150/153/195/268	32	68	54	46	0.0197	29	71	44	56	0.0275
148	At1g76510	ARID/BRIGHT DNA-binding domain-containing protein	189/212	28	72	16	84	0.0073	37	63	25	75	0.0006
149	At2g27230	Transcription factor-related	208/246	28	72	18	82	0.0177	34	66	22	78	0.0060
189	At5g43270	SPL2 (squamosa promoter binding protein-like 2); DNA binding/transcription factor	160/244	87	13	63	37	0.0021	87	13	69	31	0.0044
261	At4g10100	CNX7/SIR5; catalytic	254/270	58	42	46	54	0.0007	58	42	45	55	0.0028
272	At3g23900	RNA recognition motif (RRM)-containing protein	118/125	30	70	22	78	0.0008	30	70	23	77	0.0041
285	At3g19840	FF domain-containing protein/WW domain-containing protein	171/207	61	39	40	60	0.0004	65	35	43	57	0.0084
322	At2g33480	ANAC041 (Arabidopsis NAC domain containing protein 41); transcription factor	321/399	17	83	34	66	0.0047	15	85	29	71	0.0025
324	At5g43270	SPL2 (squamosa promoter binding protein-like 2); DNA binding/transcription factor	186/270	86	14	66	34	0.0046	95	5	66	34	0.0158
343	At3g29160	AKIN11 (Arabidopsis snf1 kinase homolog 11); protein kinase	159/307	30	70	3	97	0.0008	45	55	2	98	0.0009
Exon skipping													
181	At5g05550	Transcription factor	210/308	64	36	74	26	0.0143	59	41	75	25	0.0044
196	At3g01150	PTB (polypyrimidine tract-binding); RNA binding	165/268	91	9	97	3	0.0035	92	8	97	3	0.0071
226	At4g24740	AFC2 (Arabidopsis fus3-complementing gene 1); kinase	143/309	19	81	47	53	0.0020	12	88	45	55	0.0004
227	At4g24740	AFC2 (Arabidopsis fus3-complementing gene 1); kinase	152/343	30	70	73	27	0.0000	33	67	68	32	0.0005
380	At5g08185	npcRNA 78; MIR162a	103/168	64	36	42	58	0.0044	83	17	75	25	0.0267
Intron retention													
327	At5g59950	RNA and export factor-binding protein, putative	226/422	84	16	50	50	0.0020	93	7	69	31	0.0091

The two AS isoforms considered here are underlined.

AS pairs that only change in one of the *At*GRP7-ox lines could either be false targets or undergo differential splicing in the two ecotypes themselves. When the AS ratios were directly compared between the Col-2 and C24 wt plants 61 of the 87 AS events showed no significant changes. On the other hand, 26 AS events showed significant differences (>5%; *P* < 0.05) between Col-2 and C24 wt (Supplementary Table S4). This differential splicing could be due to sequence variation (SNPs and indels) in splice sites, in other signals which directly affect the splicing of specific introns, and in primer binding sites or could be due to differential expression of *trans*-acting factors. To determine whether differences in AS in the two *At*GRP7-ox lines could be explained by ecotype differences, we compared the sequences surrounding the splicing events (position −50 to +50 relative to the splice sites) between the two ecotypes (46).

In some cases, local sequence variation was detected that could potentially contribute to the different splicing patterns observed. For example, two primer pairs (#51 and #105) did not generate RT-PCR products in the C24 background and both had mismatches in one of the primers. For two primer pairs (#160 and #270), a transcript isoform observed in Col-2 was absent in C24. The sequence of the alternative 5′ splice site in #270 has a G to C mutation at position −1 in C24 that may be responsible for the difference in usage of this site between ecotypes (Supplementary Figure S2A). In contrast, for #160 there are no SNPs in the splice site sequences but there are sequence differences in the surrounding intron (Supplementary Figure S2B). Four primer pairs detected a significant difference in AS between ecotypes and in response to *At*GRP7 over-expression (#72, #118, #128 and #343) (Supplementary Figure S2C–F). In all four cases, the effect of *At*GRP7 over-expression was in the same direction in both backgrounds despite the different pattern in the two ecotypes. Primer pairs #171 and #179 showed differences between Col-2 and C24, and over-expression of *At*GRP7 in Col-2 background shifts the pattern in the direction of the C24 wt situation. Over-expression of *At*GRP7 in C24 had a smaller effect on the already elevated levels in C24 (Supplementary Figure S2G and S2H).

Overall, the majority of genes did not show significant variation in AS ratios between the two ecotypes. Where significant qualitative or quantitative variation was observed, SNPs and indels in or near splice sites may explain the differential splicing behavior.

### *At*GRP7 and *At*GRP8 impact an overlapping set of AS events

*At*GRP7 cross-regulates its paralog *At*GRP8 that is 77% identical in sequence (31,40). Constitutive over-expression of *At*GRP8, in turn, promotes production of the NMD-sensitive AS isoform of *AtGRP7*. Thus, *At*GRP7 and *At*GRP8 are able to bind their own and each others’ pre-mRNAs and may act similarly on downstream targets. We analyzed this by monitoring the impact of *At*GRP8 over-expression on 87 events including the events most strongly affected in *At*GRP7-ox (Col-2) plants. A total of 30 AS events showed a significant change in *At*GRP8-ox plants including *AtGRP7* AS at the cryptic 5′ splice site within its intron (Figure 1B). Of these, 27 also showed a change in *At*GRP7-ox (Col-2) and over-expression of either *At*GRP7 or *At*GRP8 affected these AS events in the same direction (Supplementary Table S5). Thus, *At*GRP7 and *At*GRP8 not only cross-regulate but also share a number of downstream targets.

### Inverse regulation of AS events by *At*GRP7 gain-of-function and loss-of-function

The genes showing changes in AS in *At*GRP7-ox plants may be direct targets of *At*GRP7 or indirect targets whose changes in AS are due to the altered expression of splicing regulators. We reasoned that if a gene is a direct target the AS events should also respond to reduced levels of *At*GRP7 and, moreover, the ratio of the AS forms should change in the opposite direction.

In the *atgrp7-1 *T-DNA insertion line that lacks *At*GRP7 (41), steady-state abundance of *At*GRP8 is strongly elevated (39). This is most likely due to relief from repression, as over-expression lines of *At*GRP7 show little *AtGRP8* expression (31,40). There is no true loss-of-function mutant in *At*GRP8 that has been characterized to date. To obtain plants that lack *At*GRP7 and express reduced amounts of *At*GRP8, *atgrp7-1* was crossed to the RNAi line *AtGRP8*i-l71 (39). F2 plants were identified that show an *At*GRP8 protein level close to wt, indicating that the RNAi construct substantially reduced the increased *At*GRP8 level in *atgrp7-1* (Supplementary Figure S1). In this line, designated *atgrp7-1 8i*, 17 of the 87 investigated AS events (20%) changed significantly by >5% (*P* < 0.05) (Figure 1C). Thus, fewer AS events were affected by the reduced *At*GRP7 level than when *At*GRP7 was over-expressed. For 10 of the events that changed in both *At*GRP7-ox (Col-2) and *At*GRP7-ox (C24), as well as in the *atgrp7-1 8i* line, the ratio of AS isoforms in *atgrp7-1 8i* changed significantly in the opposite direction to *At*GRP7-ox plants, suggesting these transcripts may be direct targets of *At*GRP7 (Table 3). An additional five events showed a smaller, yet statistically significant change between 3% and 4.75% in the opposite direction (*P* < 0.05) (Table 3).

**Table 3.** Genes/transcripts with changes in the AS pattern in opposite direction in *At*GRP7-ox and *atgrp7-1 8i* plants

Primer pair	AGI	Description	Product sizes (bp)	*grp7-1 8i*		*P*-value *grp7-1 8i*	Col-2		*At*GRP7-ox (Col-2)		*P*-value 7-ox (Col-2)	*At*GRP8-ox (Col-2)		*P*-value 8-ox (Col-2)
				Small product (%)	Large product (%)		Small product (%)	Large product (%)	Small product (%)	Large product (%)		Small product (%)	Large product (%)	
12	At1g72320	APUM23 (Arabidopsis pumilio 23); RNA binding	141/150/215	21	77	0.0001	15	83	5	95	0.0002	7	93	0.0099
19	At2g32320	tRNA His guanylyltransferase	202/273	27	73	0.0051	33	67	45	55	0.0047	35	65	0.2379
288	At3g12570	FYD	159/188	33	67	0.0111	43	57	52	48	0.0148	44	56	0.5852
75	At2g36000	mitochondrial transcription termination factor-related/mTERF-related	150/254	40	60	0.0076	31	69	14	86	0.0009	22	78	0.0098
90	At4g39260	ATGRP8/GR-RBP8 (CCR1)	158/316	99	1	0.0060	94	6	23	77	0.0000	98	2	0.0215
343	At3g29160	AKIN11 (Arabidopsis snf1 kinase homolog 11); protein kinase	159/307	57	43	0.0001	30	70	3	97	0.0008	11	89	0.0027
181	At5g05550	transcription factor	210/308	50	50	0.0049	64	36	74	26	0.0143	67	33	0.2237
226	At4g24740	AFC2 (Arabidopsis fus3-complementing gene 1); kinase	143/309	7	93	0.0004	19	81	47	53	0.0020	33	67	0.0506
227	At4g24740	AFC2 (Arabidopsis fus3-complementing gene 1); kinase	152/343	22	78	0.0125	30	70	73	27	0.0000	53	47	0.0155
327	At5g59950	RNA and export factor-binding protein, putative	226/422	90	10	0.0102	84	16	50	50	0.0020	68	32	0.0456
129	At2g40830	RHC1A (RING-H2 finger C1A); protein binding/zinc ion binding	220/329	94	6	0.0441	91	9	59	41	0.0012	76	24	0.0648
171	At5g18620	CHR17 (chromatin remodeling factor17); DNA-dependent ATPase	213/222	75	25	0.0179	72	28	66	34	0.0005	71	29	0.4350
273	At3g07810	heterogeneous nuclear ribonucleoprotein, putative/hnRNP, putative	126/173	12	88	0.0184	8	92	3	97	0.0094	5	95	0.0108
295	At2g02390	ATGSTZ1 (glutathione s-transferase 18); glutathione transferase	181/202	91	9	0.0165	88	12	69	31	0.0023	74	26	0.0512
322	At2g33480	ANAC041 (Arabidopsis NAC domain containing protein 41); transcription factor	321/399	12	88	0.0226	17	83	34	66	0.0047	26	74	0.0836

The first 10 events show a significant change (>5%, *P* < 0.05). For primer pair #12, the two splice forms considered are underlined (the 141 nt form does not change). The last five events show a smaller, yet statistically significant change between 2.9% and 4.75% (*P* < 0.05) in *atgrp7-1 8i* plants in the opposite direction to *At*GRP7-ox plants.

**Figure 2.**
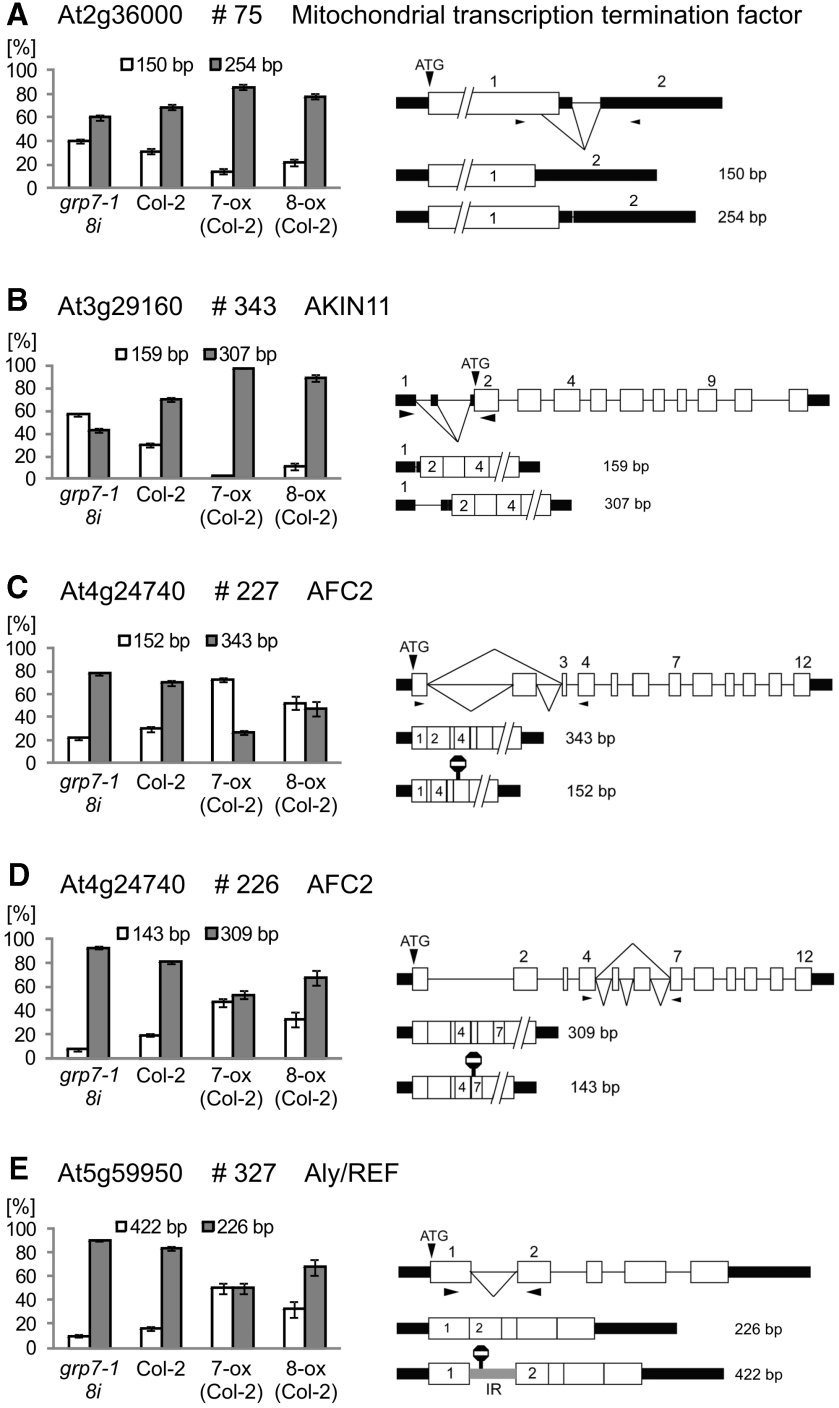
Genes/transcripts with significant changes in the AS patterns in opposite directions in *atgrp7-1 8i* and *At*GRP7-ox plants. (**A**) At2g3600 (#75) Mitochondrial transcription termination factor, (**B**) At3g29160 (#343) AKIN11, (**C**) At4g24740 (#227) AFC2, (**D**) At4g24740 (#226) AFC2 and (**E**) At5g59950 (#327) Aly/REF-like protein. On the left side of each panel, the percentage of each splice form ± SD based on three biological replicates is indicated for *atgrp7-1 8i*, wt, and *At*GRP7-ox plants, respectively. For comparison, the data for *At*GRP8-ox plants are included. On the right side of each panel, the gene and transcript structures and the AS events are shown schematically. Exons are indicated by open boxes and numbered, UTRs—black rectangles, introns—thin lines, splicing events—diagonal lines and stop signs—PTCs. The sizes of the PCR products from each splice isoform are indicated.

The three lines, *atgrp7-1 8i*, Col-2 wt and *At*GRP7-ox represent a series of genotypes with increasing amounts of *At*GRP7, and where the *At*GRP8 level is effectively the same in wt and *atgrp7-1 8i* lines and *At*GRP8 is virtually absent in the *At*GRP7-ox line (Supplementary Figure S1). In these lines, we observed reciprocal changes in the abundance of AS isoforms (Figure 2 and Supplementary Figure S3). For example, in At2g36000, encoding a mitochondrial transcription termination factor-related protein (#75), there is AS of an intron in the 3′-UTR and lower levels of *At*GRP7 give increased usage of the alternative 5′ splice site towards the end of the coding region and therefore increased levels of the shorter isoform, which could generate a C-terminally truncated protein (Figure 2A). For *AKIN11* encoding a catalytic subunit of Snf1-related (SnRK1) protein kinase (#343), an increasing *At*GRP7 concentration shifts the ratio in favor of a longer splice variant containing part of intron 1 due to enhanced usage of an alternative 5′ splice site in the 5′-UTR (Figure 2B). *AFC2* encoding a LAMMER kinase has two different AS events: skipping of exon 2 and skipping of exons 5 and 6. Increased *At*GRP7 levels cause increased skipping of exon 2 (#227), which leads to an AS isoform containing a PTC (Figure 2C). For the event detected with primer pair #226, in *At*GRP7-ox plants, both isoforms are produced at similar levels, whereas in plants lacking *At*GRP7 or wt plants, the variant with exons 4 through 7 predominates (Figure 2D). Thus, *At*GRP7 promotes skipping of exon 2 and exons 5 and 6. For At5g59950 encoding an Aly/REF-related RNA-binding protein/export factor (#327), *At*GRP7 strongly increases the proportion of the AS isoform retaining the first intron leading to introduction of a PTC (Figure 2E). For all these events, overexpression of *At*GRP8 has a similar effect as overexpression of *At*GRP7 (Figure 2 and Supplementary Figure S3). Taken together, we observed an increase of one AS isoform and the concomitant reduction of the other isoform dependent on the *At*GRP7 dosage, and in several cases, a switch in the predominant splice form occurred between plants lacking *At*GRP7 and *At*GRP7-ox plants.

### RNA immunoprecipitation shows direct binding of *At*GRP7 to target transcripts *in vivo*

**Figure 3.**
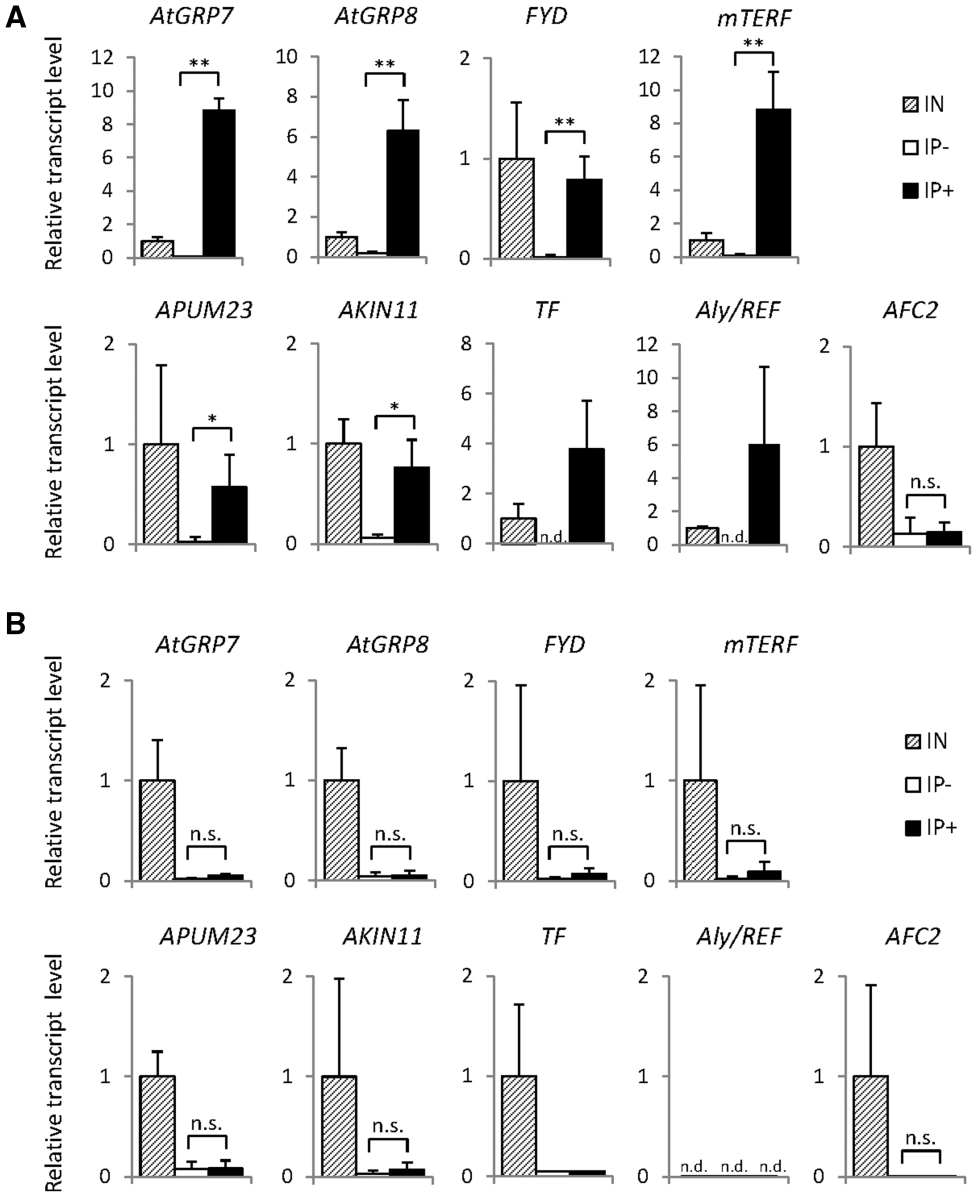
*In vivo* interaction of *At*GRP7-GFP with candidate target transcripts. RIP was performed on plants expressing the *At*GRP7-GFP fusion protein under control of the *AtGRP7* promoter including 5′- and 3′-UTR and intron in *atgrp7-1* (**A**) and transgenic plants expressing GFP under control of the *AtGRP7* promoter including 5′- and 3′-UTR (**B**). The levels of transcripts co-precipitated in the GFP-Trap® bead precipitate (IP+), the RFP-Trap® bead mock precipitate (IP−) and in the input fractions, respectively, were determined by qRT-PCR in duplicates for At2g21660 (*AtGRP7*) which served as positive control, At4g39260 (*AtGRP8*, #90), At3g12570 (*FYD*, #288), At2g3600 (*mTERF*, #75), At1g72320 (*APUM23*, #12), At3g29160 (*AKIN11*, #343), At5g05550 [transcription factor (*TF*), #181], At4g24740 (*AFC2*, #226 and #227) and At5g59950 (*Aly/REF*-like RNA binding/export factor, #327). Transcript levels were normalized to *PP2A* and expressed relative to the input. Means ± SD are presented based on three biological and significance was tested using Student’s *t*-test (**P < 0.005, *P < 0.05). n.d., not detectable; n.s., not significant. In IP− from GFP plants, the transcripts #181 and #327 were not detectable and thus no statistical test was applied.

The antagonistic regulation in *At*GRP7 loss-of-function and gain-of-function lines, respectively, suggests that *At*GRP7 may directly interact with the affected transcripts. To study the potential *in vivo* association of the transcripts with *At*GRP7, we established an efficient protocol to immunoprecipitate ribonucleoprotein complexes from whole cell extracts, followed by qPCR. An *At*GRP7-GFP fusion protein driven by the *AtGRP7* promoter and all regulatory elements in the transcribed region was expressed in the *atgrp7-1* background. As proof-of-principle, we showed that the *AtGRP7* transcript, a known *in vitro* binding substrate of *At*GRP7 was efficiently precipitated with GFP-Trap® beads (IP+) but was barely detected in mock precipitates using RFP-Trap® beads (IP−) (Figure 3A). As an additional control, we performed RIP on plants expressing GFP only driven by the same regulatory elements. Only a very small amount of *AtGRP7* was detected in the IP+ fraction relative to the input, and no enrichment was detected relative to IP− (Figure 3B). This demonstrates that the procedure faithfully identifies a known binding substrate.

We next examined the precipitated RNA for the presence of the candidate targets (depicted in Figure 2 and Supplementary Figure S3). The *At*GRP8 transcript (#90), the transcript of the mitochondrial transcription termination factor-related protein (*mTERF*, #75), and the *FYD* transcript (#288) were strongly enriched in the IP+ fraction but not in the mock precipitate (IP−) (*P* < 0.005) (Figure 3A). In the GFP plants, a far lower level relative to input and no difference between IP+ and IP− were detected (Figure 3B). *APUM23* encoding a member of the Pumilio RBP family (#12) and *AKIN11* (#343) showed a weaker yet statistically significant enrichment in IP+ versus IP− from *At*GRP7-GFP plants (Figure 3A) (*P* < 0.05), and no enrichment in IP+ of GFP plants (Figure 3B). The transcript encoding a transcription factor (TF, #181) was enriched in IP+ but detected only once in IP−. The Aly/REF-like protein (#327) was enriched in IP+ but not detectable in IP− and the GFP plants. In several cases, levels of transcripts coprecipitated with *At*GRP7-GFP appeared higher than the input level. This has been observed before and may be attributed to higher efficiency of RNA extraction and amplification from the IP compared to the total extract (47,48).

Although *AFC2* (#226 and #227) was recovered from *At*GRP7-GFP plants but not from GFP plants, the signals in IP+ and IP− were similar and therefore *AFC* either may not be a specific *in vivo* substrate of *At*GRP7 or the interaction was too weak to be detected. The expression level of At2g32330 encoding an unknown protein (#19) was too low to allow a reliable quantification. Thus, using RIP-qPCR, we were able to confirm direct binding of *At*GRP7 to seven putative target transcripts identified by their antagonistic AS behavior in the over-expression lines and the loss-of-function mutant.

### Site-specific mutation of the conserved Arg^49^ in RNP-1 abrogates the effect of *At*GRP7 on AS events

**Figure 4.**
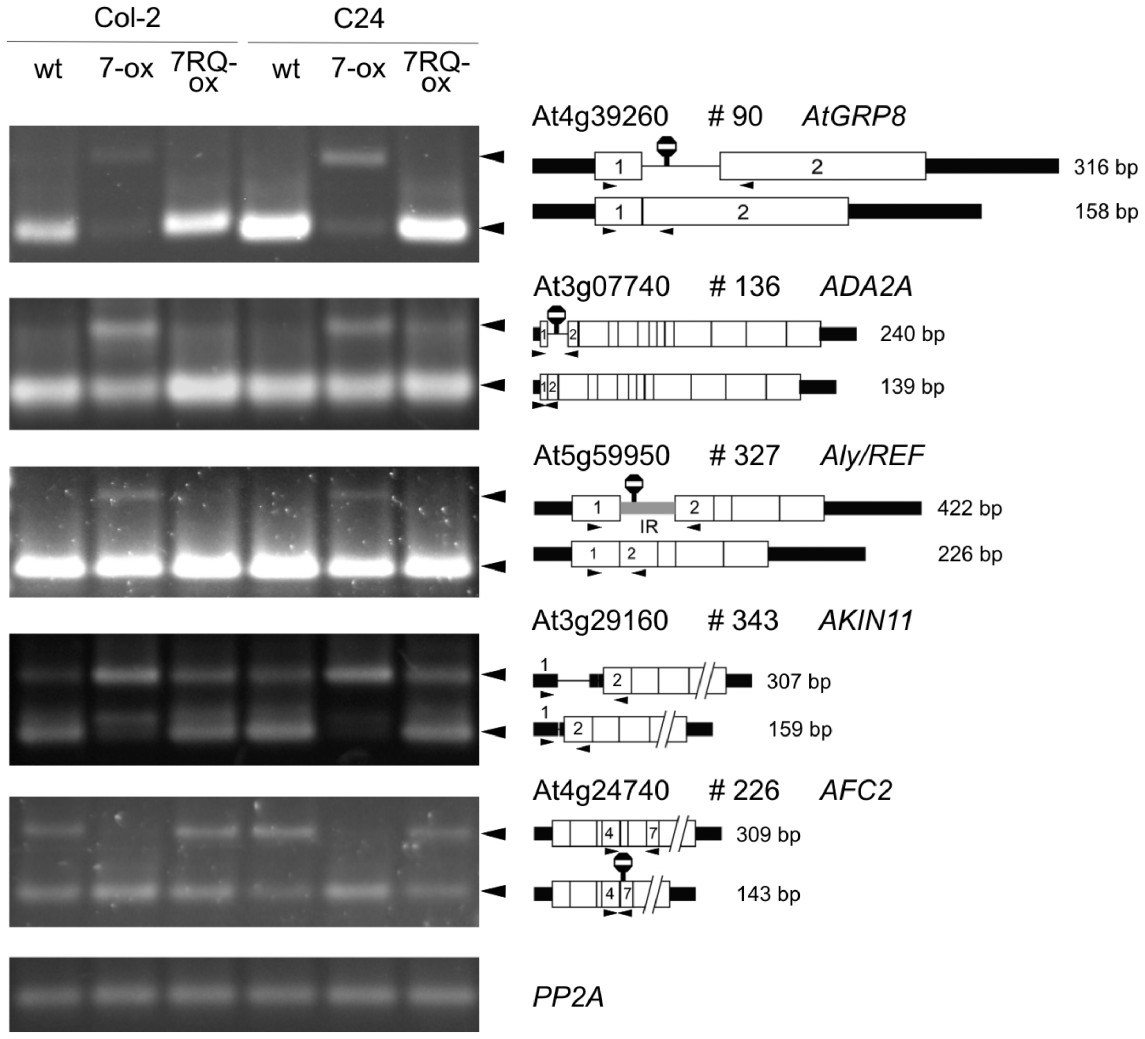
AS events in *At*GRP7-ox lines not observed in *At*GRP7-R^49^Q-ox plants. Left side: RT-PCR analysis of selected genes/transcripts with total RNA from *At*GRP7-ox and *At*GRP7-RQ-ox plants in both the Col-2 and C24 backgrounds and their respective wt plants. Arrowheads denote RT-PCR products representing AS events whose presence/absence or abundance differs between the over-expression lines with and without the R^49^Q mutation. Right side: gene and transcript structures and the AS events for *At*GRP8 (#90), *ADA2A* (#136), Aly/Ref (#327), *AKIN11* (#343) and AFC2 (#226) are shown schematically (see Figure 2). *PP2A* served as constitutive control.

Mutation of a single arginine to glutamine within the RRM interferes with both *in vitro* binding of recombinant *At*GRP7 to its own RNA and the impact of *At*GRP7 on *AtGRP7* and *AtGRP8* pre-mRNA splicing *in vivo* (36). Therefore, we investigated the splicing pattern of selected candidate *At*GRP7 targets in two independent transgenic lines constitutively over-expressing the mutant protein (*At*GRP7-RQ-ox). As a control, we showed that over-expression of *At*GRP7 affected AS of *AtGRP8* by causing part of the intron to remain in the transcript, generating the NMD-sensitive AS isoform (Figure 4). This *At*GRP7-induced change in AS of *AtGRP8* was not observed in *At*GRP7-RQ-ox plants (36).

For the transcriptional activator adapter *ADA2A* (#136), ectopic *At*GRP7 expression increased the proportion of the larger splice variant compared to wt plants through increased use of an alternative 5′ splice site and inclusion of part of the intron. This effect was not seen upon ectopic expression of *At*GRP7-RQ. Similarly, the larger splice form of the Aly/REF-related protein (#327) that retains the first intron appeared in *At*GRP7-ox plants but not in *At*GRP7-RQ-ox plants. For *AKIN11* (# 343), over-expression of *At*GRP7 wt protein led almost exclusively to the production of the long splice variant retaining the intron in the 5′-UTR, whereas over-expression of *At*GRP7-RQ resembled wt plants. Finally, for the LAMMER kinase *AFC2* (#226), the transcript where exons 5 and 6 are skipped was detected almost exclusively in *At*GRP7-ox plants but not in *At*GRP7-RQ-ox plants. Thus, in all of these examples, the effects observed with over-expression of authentic *At*GRP7 protein are lost when the *At*GRP7-RQ mutant protein is over-expressed suggesting that AS of these genes depends on the RNA-binding activity of *At*GRP7.

Based on these results, we investigated further AS events that showed opposite splicing behavior in *At*GRP7-ox and *atgrp7 8i* lines (Table 3) in the *At*GRP7-RQ-ox plants using the RT-PCR panel. None of the 14 analyzed events showed a difference in AS pattern in wt and the *At*GRP7-RQ-ox line in Col-2, again suggesting that these events are also dependent on the conserved arginine in the RRM (data not shown).

### The influence of *At*GRP7 on AS events generating NMD substrates

AS can regulate expression levels by generating transcript isoforms that are degraded by the NMD pathway. In *Arabidopsis*, ∼13–18% of transcripts are estimated to undergo AS-linked NMD by identifying AS forms stabilized in *upf1-5* and *upf3-1* mutants or upon cycloheximide (CHX) treatment of wt plants using the high resolution RT-PCR system also used here (35). Because *At*GRP7 and *At*GRP8 auto- and cross-regulate by binding to their pre-mRNAs to generate an NMD-sensitive AS transcript (31), we mined the data by Kalyna *et al.* for the behavior of the 10 pairs of splice isoforms that showed mirror-image patterns in plants with high *At*GRP7 level and plants that lack *At*GRP7, respectively. Seven of these events included a PTC in one AS isoform (Supplementary Table S6). In six cases, At2g32330 (unknown protein; #19), mTERF-related protein (#75), *At*GRP8 (#90), AFC2 (#226 and #227) and Aly/REF-related protein (#327), elevated levels of *At*GRP7 favored the production of the PTC-containing form. In four of these cases (#19, #90, #226 and #327) the PTC-containing form is stabilized in *upf* mutants and/or upon CHX treatment (35). For #181, the non-PTC-containing form was favored by increased levels of *At*GRP7. The other three AS events with opposite AS patterns involved AS in introns in the 5′-UTR and gave rise to AS isoforms that were stabilized in *upf3-1* (#12), in *upf3-1* and upon CHX treatment (#343) or only upon CHX treatment (#288), respectively, although no obvious effect of an upstream open reading frame or NMD signals were found (35). Thus, *At*GRP7 regulates the expression of, at least, some of its target genes by affecting NMD-sensitive AS isoforms.

## DISCUSSION

Here, we identify the hnRNP-like proteins, *At*GRP7 and *At*GRP8, as novel splicing regulators in *Arabidopsis* using a high resolution RT-PCR system capable of detecting changes in AS. Constitutive over-expression of *At*GRP7 caused significant changes in the ratio of AS isoforms in 21% of the 288 investigated AS events.

To study the effect on AS of *At*GRP7 in detail, we used a series of lines (over-expression, wt and loss-of-function mutant) expressing *At*GRP7 at different levels. Generation of the *At*GRP7 knock-down was complicated by up-regulation of *At*GRP8 in the *atgrp7-1* mutant (39) and required it to be combined with an RNAi line reducing *At*GRP8 expression. This genotype, *atgrp7-1 8i*, allowed us to study the effect of loss of *At*GRP7 with *At*GRP8 expression at a similar level to wt plants (Supplementary Figure S1). The advantage of comparing lines with different levels of *At*GRP7, effectively representing a dosage series, became apparent when over-expression and loss-of-function of *At*GRP7 had opposite consequences on 10 AS events, suggesting that these transcripts represent direct targets. Importantly, RIP from whole cell extracts confirmed that seven of the transcripts are indeed bound by *At*GRP7 *in vivo*. Thus, dosage-dependent splicing behavior acts as an indicator of direct interaction of proteins affecting AS site choice. In line with this, the events with a reciprocal change in the AS ratio upon increasing or decreasing *At*GRP7 levels were influenced by over-expression of the *At*GRP7 wt protein but not of the mutant version where arginine of RNP-1 was exchanged for glutamine (R^49^Q). This mutation has been shown to impair the RNA-binding activity of recombinant *At*GRP7 and the *in vivo* function (36,49). Thus, it is a valuable tool for future studies of the physiological consequences of *At*GRP7 mis-expression on splicing of target genes identified here. Initial experiments to identify conserved sequence motifs surrounding the AS events in the direct *At*GRP7 targets were not successful, presumably due to the small sample size. Moreover, current computational programs to identify conserved sequence motifs at the RNA level still have their limitations: In addition to the sequence context, structural features of the RNA are relevant (50).

In contrast to the 10 events with reciprocal changes, 3 AS events showed a significant change in the ratio of AS forms in the same direction both in *atgrp7-1 8i* and *At*GRP7-ox plants (Supplementary Table S5). The identical effect of perturbation of *At*GRP7 steady-state abundance in either direction could indicate that these AS events are controlled by a protein complex involving *At*GRP7.

Loss of *At*GRP7 affected fewer transcripts than elevated levels. Thus, *At*GRP7 is clearly required for normal regulation of some AS events but is not limiting for others. This is likely to be due to functional redundancy with other splicing regulators such as other hnRNP proteins and, in particular, *At*GRP8 that is still expressed at a low level in *atgrp7-1 8i*. Indeed, the interdependence of *At*GRP7 and *At*GRP8 makes it difficult to disentangle the effect of the paralogs on AS of downstream transcripts. Here, we show a clear overlap in AS of target transcripts which would be consistent with these proteins having similar RNA-binding properties (51,52). However, *At*GRP7 also affected pre-mRNAs uniquely suggesting that its role in splice site selection is modulated by interactions with other proteins. Another feature of the interaction of *At*GRP7 with pre-mRNAs is the over-representation of events involving alternative 5′ splice sites in those affected by *At*GRP7. This observation is consistent with our previous results showing that elevated levels of *At*GRP7 led to preferential use of an alternative 5′ splice site both within the *AtGRP7* and *AtGRP8* intron (30,31).

*At*GRP7 and *At*GRP8 levels are regulated by linked AS and NMD. For 7 of the 10 AS events with opposite splicing patterns in *At*GRP7-ox and *atgrp7-1 8i*, a recent study has found that one of the splice isoforms is stabilized in *upf* mutants impaired in NMD and/or upon CHX treatment of wt plants (35). Although only a small sample of genes/transcripts, the enrichment of NMD substrates among *At*GRP7 targets suggests that the impact of *At*GRP7 on splicing events has functional consequences.

Finally, in corroborating putative AS targets of *At*GRP7, we used independent over-expression lines in two different genetic backgrounds and found that most events were influenced by *At*GRP7 over-expression in both ecotypes. Comparison of Col-2 and C24 wt plants showed that 70% of events were unaffected by the ecotype background. This demonstrates that the HR RT-PCR system can be useful in the analysis of splicing factor mutants even if these are in different ecotypes. On the contrary, almost 30% of the AS events showed significant changes (>5%, *P* < 0.05) between the two ecotypes. Many of these AS events had sequence variation in the form of SNPs/indels in the region of the splicing events. Thus, the panel can in turn complement transcriptome analysis of *Arabidopsis* accessions by high-throughput sequencing for detecting ecotype variation in AS (10,53). As the identified SNPs did not affect the splice site consensus sequences themselves, further systematic analysis of the SNPs/indels and their qualitative and quantitative effects on AS will be required to elucidate the impact of particular sequences in different sequence contexts and improve predictability of consequences of sequence variation.

### Overlapping targets with other factors influencing AS

Splice site selection and assembly of the spliceosome depends on the recognition of sequences in the pre-mRNA by multiple protein factors and interactions between them. In humans, genome-wide mapping of human splicing regulatory proteins to their target RNA sequences is generating a ‘splicing code’ that will ultimately allow prediction of AS behavior of transcripts in different cells and tissues (8,18,54,55). For the most part, splicing factors will affect AS of numerous downstream target pre-mRNAs and their function will be modulated by interactions with other factors bound to the same pre-mRNA such that splicing behavior will be determined by the relative abundance and activity of different factors within a network of AS regulation (1). In plants, our knowledge of the functions of factors which affect AS is relatively limited particularly in terms of their binding sites and interactions (56–58). Information on the effects on splicing and AS of individual genes is increasing particularly for SR proteins but also for hnRNP proteins such as the PTB family, *At*GRP7 and *At*GRP8, and other RBPs (12,19,21,22,59–61) but virtually nothing is known about common targets of different factors.

The HR RT-PCR system has been used to examine the effects on AS of SR30, At-RS2Z33 (RSZ33) and cap-binding complex (CBC) proteins in addition to *At*GRP7 and *At*GRP8 (33,34). AS of RSZ33 (#21) was influenced in the same direction by over-expression of At-RS2Z33, SR30 and *At*GRP7, respectively, leading to an AS form that does not produce functional protein (Supplementary Tables S2 and S5). Over-expression of SR30 altered splice site usage in At1g04400 (cryptochrome2, #2), At2g32320 (tRNA His Guanylyltransferase #19), At4g12790 (ATP-binding family protein, #36), At5g04430 (KH domain-containing protein NOVA, #42) in the same direction as over-expression of *At*GRP7, and in At5g41150 (UV hypersensitive 1, #49) and At5g66010 (#59, unknown RNA-binding protein) in the opposite direction. Thus, we show here both an effect in the same direction and an antagonistic effect of SR proteins and the hnRNP-like *At*GRP7, as often observed in mammals.

Mutants defective in components of the CBC also significantly affected 101 of 252 analyzed AS events (>3% change; *P* ≤ 0.1), implicating the CBC in the choice of AS sites (34). In the absence of complementary data on gain-of-function mutants or *in vivo* binding data, it is not yet clear which transcripts are direct targets. Notably, the CBC, like *At*GRP7, also preferentially influences alternative 5′ splice site selection. Loss of CBP20 and/or CBP80 and the loss of *At*GRP7 in *atgrp7-1 8i* altered splicing in the same direction in the case of At2g40830 encoding a zinc finger protein (#129) and of *AFC2* (#227), suggesting that *At*GRP7 and the cap binding complex have a similar effect on these AS events. In turn, for At5g18620 encoding chromatin remodeling factor 15 (#171), FYD (#288) and AKIN11 (#343) loss of CBP20 and/or CBP80 and loss of *At*GRP7 shift the splicing ratio in the opposite direction, suggesting that *At*GRP7 acts antagonistically to the CBC on these AS events.

Many splicing factors in animals and plants are themselves regulated at the level of AS often involving conserved splice site sequences (62–64). AS of splicing factors that are involved in determining AS of other transcripts generates a hierarchical network of splicing regulators influencing downstream targets. For example, CBPs affect AS of PTB1, RS2Z33 and SR30 (34). Here, we show that *At*GRP7 affects AS of transcripts encoding putative RBPs and predicted splicing regulators (Table 3), among those PTB1, RS2Z33 and SR30 which in turn influence splice selection of other targets. Among the targets of *At*GRP7 is *AFC2* encoding a LAMMER kinase. LAMMER kinases share an EHLAMMERILG motif in their catalytic subdomain X, giving rise to their name. In mammals, *Drosophila* and fission yeast members of the LAMMER kinase family are involved in AS through phosphorylation of SR proteins (65–67). *Arabidopsis* AFC and the tobacco ortholog PK12 phosphorylate SR proteins *in vitro* (68,69). Furthermore, heterologous expression of PK12 in transgenic *Arabidopsis* modulates AS of specific transcripts. For example, AtSR30 has an AS event involving alternative 3′ splice sites in intron 10 (#3) that is influenced in the same way upon expression of PK12 in transgenic *Arabidopsis* and over-expression of *At*GRP7 or *At*GRP8 (Supplementary Table S5). On the other hand, *AFC2* itself is spliced into multiple AS variants, and skipping of exon 2 (#227) and of exons 5 and 6 (#226) resulting in unproductive mRNAs is promoted in *At*GRP7-ox plants, pointing to a complex interaction. Skipping of exon 2 is reduced both in the *cbp20* and *cbp20 cbp80* (34) and *atgrp7-1 8i* mutants, suggesting that *At*GRP7 and CBC both negatively impact exon2 inclusion.

**Figure 5.**
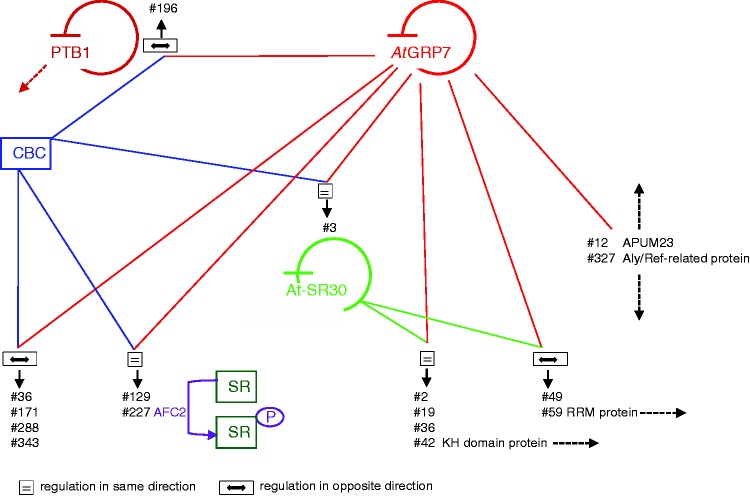
Conceptual model depicting common targets of known splicing regulators in *Arabidopsis*. The impact of *At*GRP7, the cap binding complex (CBC) and At-SR30 on selected splicing events, analyzed by the RT-PCR panel, is displayed (33,34). The numbers of the primer pairs for detection of the AS events are indicated. Regulation of the ratio of splice isoforms in the same direction is indicated by ‘=’; regulation in opposite direction is indicated by a line with two arrowheads. For RNA-binding proteins that are influenced by *At*GRP7, the name is indicated and dotted arrows indicate a presumed post-transcriptional regulation of yet unknown targets of these proteins. The negative autoregulation of *At*GRP7, PTB1 and At-SR30 is depicted (19,31,57). ‘P’ denotes phosphorylation of SR proteins by the LAMMER kinase AFC2 (68). For clarity, the effects of *At*GRP8 and At-RS2Z33 on AS events are omitted (see text for details).

In addition, ectopic *At*GRP7 expression in Col-2 promoted skipping of exon 3 in *PTB1* (#196). PTBs are hnRNP proteins that control AS of an extensive network of downstream transcripts in humans (70). In *Arabidopsis*, alternative inclusion of a cassette exon in PTB1 leads to a PTC and degradation via the NMD pathway (19) such that *At*GRP7 favored the production of functional mRNA (Supplementary Table S2). Finally, *At*GRP7 and *At*GRP8 have been identified as substrates of the protein arginine methyltransferase *At*PRMT5, an *Arabidopsis* homolog of human PRMT5 involved in methylation of histones and Sm proteins of spliceosomal small nuclear ribonucleoprotein particles (71). Mutation in *At*PRMT5 leads to splicing defects in hundreds of genes, inviting the speculation that some of the PRMT5 effects may be mediated via *At*GRP7 and/or *At*GRP8 (32). A conceptual model of the interaction of *At*GRP7 with known splicing regulators is shown in Figure 5.

In conclusion, our data indicate that the hnRNP-like proteins *At*GRP7 and *At*GRP8 are novel splicing regulators in *Arabidopsis* that affect a number of downstream targets. Both the influence of *At*GRP7 on AS of other splicing regulators or RBPs and the observation that *At*GRP7 shares targets with other splicing regulators like the CBC point to an extensive network of post-transcriptional regulation in *Arabidopsis* (18,72,73).

## SUPPLEMENTARY DATA

Supplementary Data are available at NAR Online: Supplementary Tables 1–6 and Supplementary Figures 1–3.

## FUNDING

EMBO short term fellowship (to C.S.); German Research foundation [STA 653/2, SFB613 to D.S.]; Biotechnology and Biological Sciences Research Council (BBSRC) [BB/G024979/1—European Research Area network (ERA-NET) Plant Genomics (Plant Alternative Splicing and Abiotic Stress)]; EU FP6 Programme—European Alternative Splicing Network of Excellence (EURASNET) [LSHG-CT-2005-518238]; Scottish Government Rural and Environment Science and Analytical Services division (RESAS) (to J.W.S.B.). Funding for open access charge: German Research Foundation.

*Conflict of interest statement*. None declared.

## Supplementary Material

Supplementary Data
