# Supplementary material for: An hnRNP-like RNA-binding protein affects alternative splicing by in vivo interaction with transcripts in Arabidopsis thaliana
Source: Nucleic Acids Res. 2012 Oct 5;40(22):11240–55. doi: 10.1093/nar/gks873 (PMC3526319; doi:10.1093/nar/gks873)
Supplement: Supplementary Data [file supp_40_22_11240__index.html]

An hnRNP-like RNA-binding protein affects alternative splicing by in vivo interaction with transcripts in Arabidopsis thaliana — An hnRNP-like RNA-binding protein affects alternative splicing by in vivo interaction with transcripts in Arabidopsis thaliana — Supplementary Data 

# An hnRNP-like RNA-binding protein affects alternative splicing by *in vivo* interaction with transcripts in *Arabidopsis thaliana*

## Supplementary Data

files

**Files in this Data Supplement:**

- Supplementary Data - pdf file
- Supplementary Data - xlsx file
