# Supplementary material for: An hnRNP-like RNA-binding protein affects alternative splicing by in vivo interaction with transcripts in Arabidopsis thaliana
Source: Nucleic Acids Res. 2012 Oct 5;40(22):11240–55. doi: 10.1093/nar/gks873 (PMC3526319; doi:10.1093/nar/gks873)
Supplement: Supplementary Data [file supp_gks873_nar-01746-a-2012-File008.pdf]

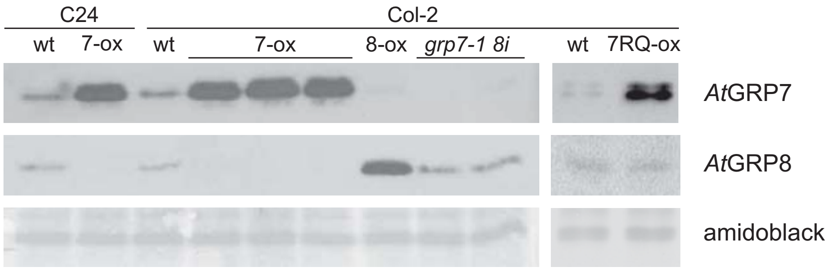

Supplementary Figure S1. Expression of *At*GRP7 and *At*GRP8 in plants employed in this study.

Protein extracts of the transgenic lines and corresponding wt plants were probed with antipeptide antibodies against *At*GRP7 and *At*GRP8, respectively. Staining of the membrane with amidoblack was used as loading control.

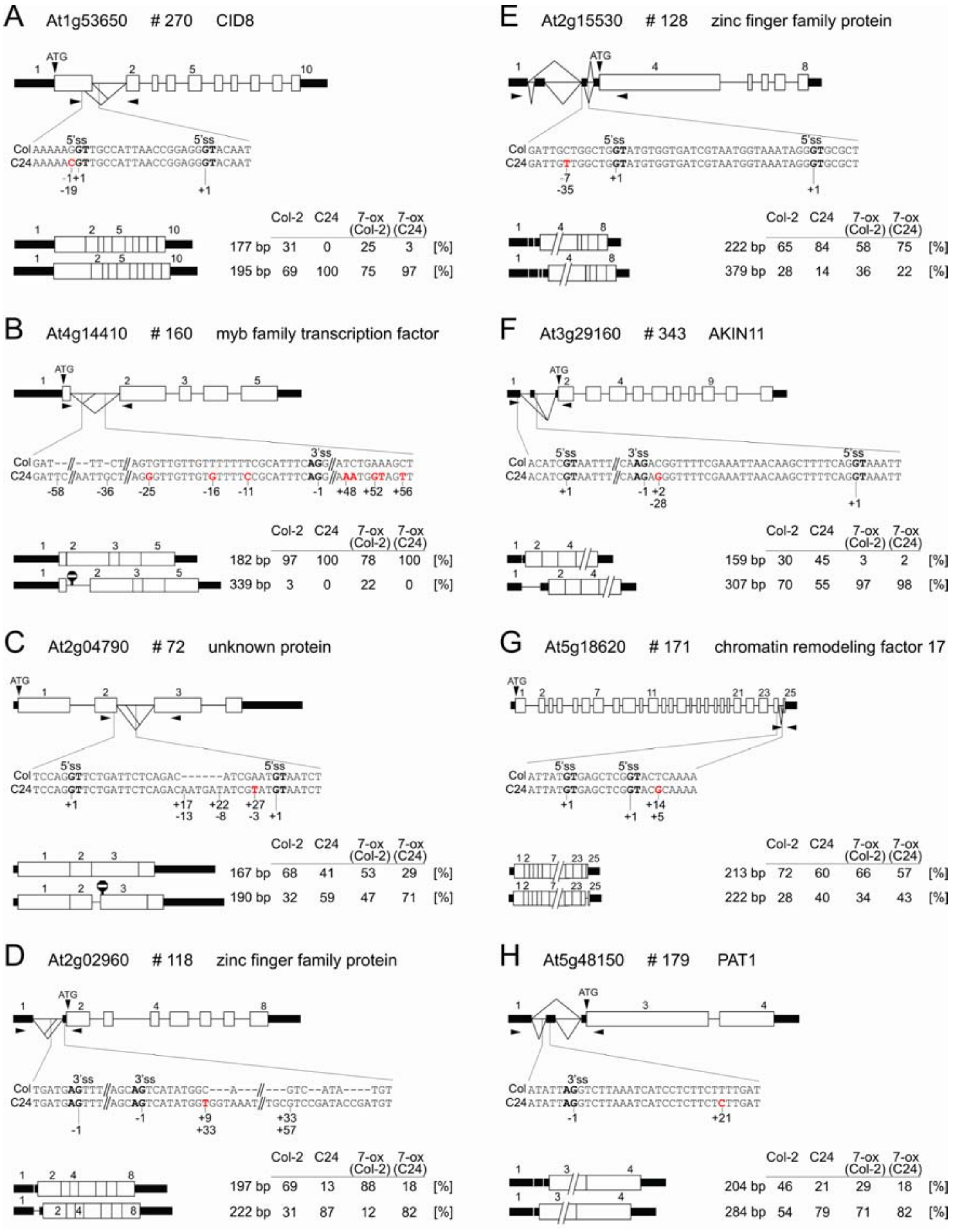

Supplementary Figure S2. Sequence variation in regions of the amplified AS events in Col-2 and C24 in genes showing significant changes between ecotypes and/or in response to *At*GRP7 over-expression.

Supplementary Figure S2. (continued)

Schematic diagrams of gene structures, amplified regions and AS events with alignment of the Col and C24 sequence and changes in AS ratios for wt and *At*GRP7-ox lines.

A) At1g53650 (#270; CID8). In Col-2, an alternative 5' splice site in exon 1 removes 18 nt to generate a shorter isoform which is absent in C24. There is a G to C mutation at -1 of the alternative site in C24.

B) At4g14410 (#160; myb transcription factor). An alternative 3' splice site in intron 1 leaves 157 nt of intron 1 in the transcript in Col-2 but this is absent in C24. There are various SNPs/indels in the surrounding intron sequence but none directly in the alternative splice site.

C) At2g04790 (#72; unknown protein). An alternative 5' splice site in intron 2 leaves 23 nt in the transcript in Col-2 but a 6 nt insertion in C24 leaves 29 nt. C24 also contains an A to T mutation at -3 of the alternative 5' splice site – the insertion and/or mutation may cause this to be used more frequently in C24.

D) At2g02960 (#118; zinc finger family protein). In Col-2, the alterative splicing of the intron in the 5' UTR of At2g02960 is complex with as many as 6 different 3' splice sites being used. Four of these sites give rise to an overlapping upstream open reading frame, a feature which appears to trigger NMD (Kalyna et al., 2012). The major products of 197 and 222 bp do not generate the overlapping upstream open reading frame. C24 contains insertions in the region between the alternative 3' splice site giving rise to the shorter product and the AUG translation start site which may cause the major change in splice site selection between Col-2 and C24.

E) At2g15530 (#128; zinc finger family protein). Alternative splicing in the 5' UTR involves the inclusion/exclusion of an 138 nt exon (exon 2) and selection of alternative 5' splice sites in exon 3/intron 3. The major products are skipping of the alternative exon (222 bp) and inclusion of this exon with use of an alternative 5' splice site which adds 19 nt (379 bp). There is a C to T mutation at position -7 relative to the upstream splice site in C24 and this site is used more frequently.

F) At3g29160 (#343; AKIN11). Alternative splicing of an intron in the 5' UTR involves an alternative exon of 29 nt. The major products are fully spliced (159 bp) or inclusion of the alternative exon with the upstream intron being unspliced (307 bp). There is a C to G mutation in C24 at position 2 of the alternative exon which could affect recognition of the exon or flanking 3' splice site affecting the relative use of the 5' splice sites.

G) At5g18620 (#171; chromatin remodeling factor 17). Alternative 5' splice sites at the 3' end of exon 24 (penultimate exon) add or remove 9 nt/3 amino acids. C24 contains a T to G SNP at position +5 of the downstream 5' splice site which may increase use of this site. *At*GRP7 promotes use of this site particularly in Col-2.

H) At5g48150 (#179; PAT1). Alternative splicing of the 5' UTR intron includes or excludes exon 2. The exon is included much more frequently in C24 and there is a T to C SNP within the exon 2 sequence in C24 which could affect a binding site for a trans-acting factor to give rise to the large difference in splicing.

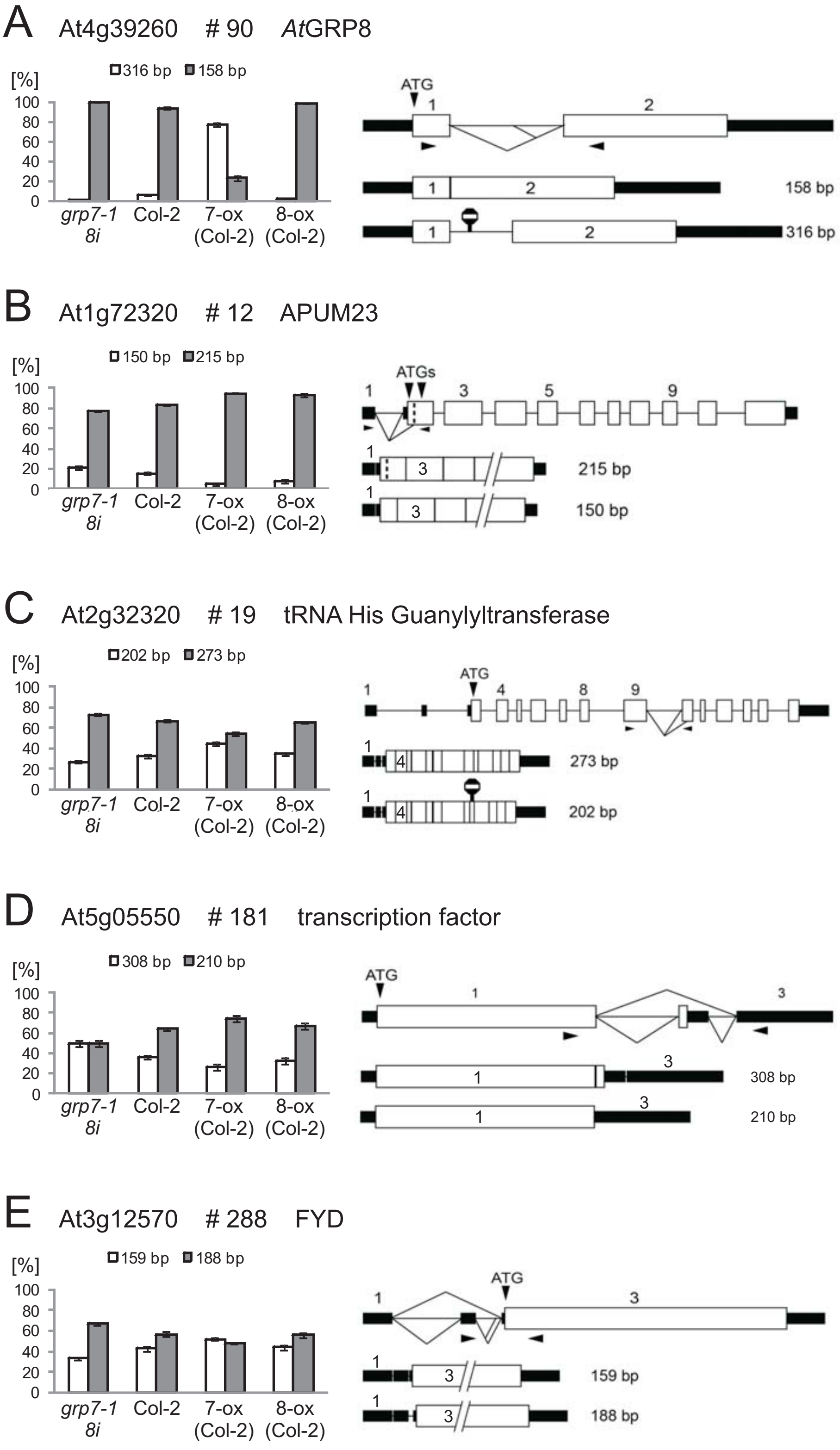

Supplementary Figure S3. Additional transcripts with significant changes in AS patterns in opposite directions in *atgrp7-1 8i* and *At*GRP7-ox plants.

Supplementary Figure S3. (continued)

A) At4g39260 (#90) (*AtGRP8*). The use of an alternative 5´splice site in the middle of the intron leads to an AS form retaining the first half of the intron including a PTC. This AS from is favored by *At*GRP7 over-expression.

B) At1g72320 (#12) (APUM23). The use of an alternative 3´ splice sites at the 5´UTR intron removes part of exon2 including the authentic ATG start codon, leading to a N-terminally truncated protein.

C) At2g32320 (#19) (tRNA His Guanylyltransferase). The use of an alternative 3´ splice site in exon 10 removes part of exon 10 and leads to a PTC.

D) At5g05550 (#181) (transcription factor). Alternative splicing of introns 2 and 3 lead to skipping of exon 2 encoding the authentic C-terminus and removal of part of the 3´UTR.

E) At3g12570 (#288) (FYD). Primers cover an alternative 3´ splice site in intron 2 in the 5´ UTR.

On the left side of each panel, the percentage of each splice form +/- s.d. based on three biological replicates is indicated for *atgrp7-1 8i*, wt, and *At*GRP7-ox plants, respectively. For comparison, the percentage of each splice form +/- s.d. in *At*GRP8-ox plants is included. On the right side of each panel, the gene and transcript structures and the alternative splicing events are indicated.
Exons are indicated by open boxes and numbered; UTRs - black rectangles; introns - thin lines; splicing events - diagonal lines; and stop signs - PTCs. The arrowheads denote the approximate position of the primers and the sizes of the PCR products from each splice isoform are indicated.
